# Correction: Kang et al. Enhanced Biomass, Paramylon, and Lipids Production by Non-Axenic Cultivation of *Euglena gracilis* in Anaerobically Digested Livestock Wastewater. *Microorganisms* 2026, *14*, 483

**DOI:** 10.3390/microorganisms14050958

**Published:** 2026-04-24

**Authors:** Yun-Ju Kang, Hyun-Jin Lim, Min-Su Kang, Yeong-Jun Lee, Jong-Hee Kwon

**Affiliations:** 1 Division of Applied Life Sciences (BK21), Gyeongsang National University, Jinju 52828, Republic of Korea; rkdbswn131@gmail.com (Y.-J.K.); nirvana970311@gmail.com (H.-J.L.); minsoo991005@gmail.com (M.-S.K.); log1124k@naver.com (Y.-J.L.); 2 Department of Food Science & Technology, Institute of Agriculture & Life Science, Gyeongsang National University, Jinju 52828, Republic of Korea

In the original publication [1], there was a mistake in the Funding and Conflicts of Interest sections. The correct Funding and Conflicts of Interest parts appear below. 

**Funding:** This research was supported by the Field Technology Research and Development Program funded by Korea South-East Power Co. (KOEN), Jinju, Republic of Korea.

**Conflicts of Interest:** The authors declare that the Korea South-East Power Co. (KOEN) company had no involvement in the study design, data collection, analysis, interpretation, the writing of this article, or the decision to submit it for publication. The authors declare that the Korea South-East Power Co. (KOEN) company had the following involvement in the study: funding.

The authors state that the scientific conclusions are unaffected. This correction was approved by the Academic Editor. The original publication has also been updated.
